# Gallbladder adhesion degree as predictor of conversion surgery, common bile duct injury and resurgery in laparoscopic cholecystectomy: A cross-sectional study

**DOI:** 10.1016/j.amsu.2021.102631

**Published:** 2021-07-30

**Authors:** Adeodatus Yuda Handaya, Victor Agastya Pramudya Werdana, Aditya Rifqi Fauzi, Joshua Andrew, Ahmad Shafa Hanif, Kevin Radinal Tjendra, Azriel Farrel Kresna Aditya

**Affiliations:** Digestive Surgery Division, Department of Surgery, Faculty of Medicine, Universitas Gadjah Mada/Dr. Sardjito Hospital, Yogyakarta 55281, Indonesia

**Keywords:** Common bile duct injury, Conversion, Gallbladder adhesion degree, Laparoscopic cholecystectomy, Resurgery

## Abstract

**Background:**

The gold-standard treatment for cholecystectomy, laparoscopic cholecystectomy, has remarkably variable outcomes and conversion rates. We investigated the gallbladder adhesion degree as a predictor of conversion surgery, common bile duct injury, and resurgery.

**Methods:**

We reviewed 157 medical records and video recordings of laparoscopic cholecystectomy on patients with cholelithiasis with or without cholecystitis at three hospitals in Yogyakarta, Indonesia from January 2016 to December 2018. The degree of gallbladder adhesion is classified into 4 categories: no adhesion, <50% adhesion, 50%-buried GB, and completely buried GB.

**Results:**

One hundred fifty seven patients were involved in this study, of whom 58 were males and 99 females with average age 49.2. Eighty-one patients out of 157 patients (51.6%) had gallbladder adhesion comprising of 61/157 (38.9%) with <50% adhesion and 20/157 (12.7%) 50%-buried GB. There is one incidence each of conversion surgery, CBD injury, and resurgery. The degree of GB adhesion has low degree of correlation with conversion surgery, CBD injury, and resurgery wirh *r* value of 0.156, 0.041, and 0.156 respectively. There is significant correlation between the degree of GB adhesion and conversion surgery and resurgery with *p* value of 0.032, and 0.032 respectively. There is no significant correlation between degree of GB adhesion and CBD injury with *p* value of 0.453.

**Conclusion:**

The degree of GB adhesion has low degree of correlation with conversion, CBD injury and resurgery. This study also showed that patients with high degree of gallbladder adhesion are still eligible for laparoscopic procedure performed by an experienced surgeon.

## Highlights

• Gallbladder adhesion is a common complication in patients with cholecystitis and cholelithiasis.
• There is low degree correlation between the degree of GB adhesion and conversion to open surgery as well as resurgery.
• There is no significant correlation between the degree of GB adhesion and common bile duct injury.

## 1 Background

Acute cholecystitis is an inflammation of the gallbladder (GB) that is most commonly caused by gallstones. Gallstones are one of the most common disorders in the gastrointestinal tract, affecting 10% of people in western countries [1]. The definitive treatment for benign gallbladder disorders is laparoscopic cholecystectomy (LC). More than 700,000 laparoscopic cholecystectomy have been completed annually in the United States since its arrival in the late 1980s [2]. According to the American College of Surgeons' National Surgical Quality Improvement Program database of 65,511 patients, nearly 90% of cholecystectomy procedures in North America are performed laparoscopically, while 10% are performed openly. Analysis of the literature revealed that the rate of conversion in laparoscopic cholecystectomy is estimated to be between 1 and 10% [3,4]. The laparoscopic technique has many benefits, including a shorter hospital stay and recovery time, less postoperative pain, and improved cosmetic outcomes [5].

Difficult laparoscopic cholecystectomy is a stressful process. The definition of (DLC) is not clearly defined, and it can vary depending on the surgeon's experience. Increased procedure time, trouble dissecting Calot's triangle or gallbladder, and complications occurring during cholecystectomy are all examples of DLC [6].

We aim to investigate adhesion as a risk factor leading to conversion from laparoscopic cholecystectomy (LC) to open surgery, common bile duct injury, and redo surgeries. This study was conceived by the first author as a surgery operator.

## 2 Methods

A retrospective study was conducted by sourcing from medical records and video recordings of patients diagnosed with cholelithiasis with/without cholecystitis who underwent laparoscopic cholecystectomy. In the duration of the study period, the surgeon performing laparoscopic cholecystectomy set up a video recording. The recordings were reevaluated by senior gastrointestinal surgeon to determine the degree of gallbladder adhesion. Subjects involved in the study were operated at three medical centers in Yogyakarta: Dr. Sardjito Hospital, Panti Rapih Hospital, and Bethesda Hospital, Indonesia from January 2016 to December 2018. These hospitals are one of the tertiary referral hospitals in Indonesia. One hundred fifty-seven patients were ascertained.

This study was approved by the Institutional Review Board of the Faculty of Medicine, Public Health and Nursing, Universitas Gadjah Mada/Dr. Sardjito Hospital, Yogyakarta, Indonesia (KE/FK/0796/EC/2018). Written informed consent was obtained from all patients for participating this study.

Adhesion degree was determined using the video recordings, and classified into no adhesion, <50% adhesion, 50% adhesion – buried GB and completely buried GB [7]. The degree was assessed by senior gastrointestinal surgeon. We evaluate three possible outcome of laparoscopic cholecystectomy that might rise due to gallbladder adhesion: surgery conversion to open cholecystectomy, common bile duct injury and resurgery.

Common bile duct (CBD) injury was determined using the intraoperative video records and assessed with Bismuth classification into 4 type: type I CBD involves distal common hepatic duct (CHD) > 2 cm from the hepatic duct confluence; type II involves the proximal CHD <2 cm from the confluence; type III involves hilar injury with no residual CHD confluence intact; type IV involves destruction of the confluence when the right and left hepatic ducts become separated and any postoperative complication related to CBD injury [8]. Surgery conversion to open cholecystectomy is defined as conversion from laparoscopic to laparotomy due to the difficulty in removing gallbladder due to adhesion. Conversion to open surgery is considered if there is no successful attempt during laparoscopic surgery within 30 min. Resurgery is done for patients who need surgical correction following previous laparoscopic cholecystectomy.

Data is presented as number and percentages for categorical variables. Chi square and Pearson correlation test was used to evaluate the correlation of different adhesion degree and conversion to open surgery, CBD injury, and resurgery. IBM SPSS Statistics version 26 (Chicago, USA) was used for statistical analysis. This paper is inline with STROCSS criteria 2019 [9].

## 3 Results

One hundred fifty-seven patients were included in the analysis comprising of 58 males and 99 females (ratio of 1:1.7). The average age of the patients was 49.2 ± 13.5 years old; the average BMI was 25.0 ± 4.5. The average of laboratories finding are: hemoglobin 13.3 ± 1.7 mg/dl; leucocyte 9.3 ± 4.5 10^3^/μL; AST 26.3 ± 26.9 U/L; ALT 31.6 ± 40.3 U/L; direct bilirubin 0.7 ± 2.5 mg/dL; indirect bilirubin 0.5 ± 1.6 mg/dL; total bilirubin 0.8 ± 1.4 mg/dL. The average length of stay was 2.85 ± 1.32 days. Based on ultrasonography examination, the percentage of patients with cholelithiasis and cholelithiasis with cholecystitis is 131/157 (83.4%) and 118/157 (75.2%) respectively. Patients are further classified into three postoperative diagnosis: cholelithiasis with single stone without cholecystitis 20/157 (12.7%), cholelithiasis with multiple stone without cholecystitis 11/157 (7%), and any cholelithiasis with cholecystitis 126/157 (80.3%) (Table 1).

**Table 1:** Baseline characteristics of laparoscopic cholecystectomy patients.

Characteristics (Unit)		Value
Sex	Male	58 (36.9%)
	Female	99 (63.1%)
Age (years)		49.2 **±** 13.5
Body Weight (kg)		65.3 **±** 14.6
Body Height (cm)		161.1 **±** 7.9
Body Mass Index (kg/m^2^)		25.0 **±** 4.5
Haemoglobin (mg/dL)		13.3 ± 1.7
Leucocyte (10^3^/μL)		9.3 ± 4.5
Neutrophil (%)		61.7 ± 13.1
AST (U/L)		26.3 ± 26.9
ALT (U/L)		31.6 ± 40.3
Direct Bilirubin (mg/dL)		0.7 ± 2.5
Indirect Bilirubin (mg/dL)		0.5 ± 1.6
Total Bilirubin (mg/dL)		0.8 ± 1.4
Cholecystitis on USG	Not Present	39 (24.8%)
	Present	118 (75.2%)
Cholelithiasis on USG	Not Present	26 (16.6%)
	Single	69 (43.9%)
	Multiple	62 (39.5%)
Postoperative Diagnosis	Single Cholelithiasis	20 (12.7%)
	Multiple Cholelithiasis	11 (7%)
	All Cholelithiasis with Cholecystitis	126 (80.3%)
Duration of Surgery (minutes)		16.2 **±** 6.3
Adhesion Degree	No	76 (48.4%)
	<50%	61 (38.9%)
	50% - Buried GB	20 (12.7%)
	Completely Buried GB	0
Surgery Conversion	Yes	1 (0.6%)
	No	156 (99.4%)
CBD Injury	Not Present	156 (99.4%)
	Type I	1 (0.6)
	Type II	0
	Type III	0
	Type IV	0
Resurgery	Yes	1 (0.6%)
	No	156 (99.4%)
Length of Stay (days)		2.85 ± 1.32

*kg = kilogram, cm = centimeter, kg/m^2^ = kilogram per meter square, mg/dL = milligram per decilitre, 10^3^/μL = 10^3^ per microliter, AST = aspartate transaminase, ALT = alanine transaminase, U/L = unit per litre, USG = ultrasonography, CBD = common bile duct.

The duration of operation averaged 16.2 **±** 6.3 min. Eighty one patients out of 157 patients (51.6%) had gallbladder adhesion comprising of 61/157 (38.9%) with <50% adhesion, 20/157 (12.7%) 50% - buried GB, and no patients with completely buried gb. There is one incidence of CBD injury (0.6% of total patients) and one incidence of conversion to open surgery (0.6% of total patients). There is one incidence of resurgery (0.6% of total patients) (Table 1).

The one patient, who is in 50% - buried GB group (5%), underwent conversion to open surgery. Analysis shows that GB adhesion degree has low degree of correlation with conversion surgery (*p* = 0.032, *r* = 0.156). One incidence of CBD injury occurs in the patient group of adhesion degree <50% (1.6%). Analysis shows no significance in the correlation between GB adhesion degree with CBD injury (*p* = 0.453, *r* = 0.041). One patient, who is in 50% - buried GB group (5%), underwent resurgery. Analysis shows that degree of GB adhesion has low degree of correlation with resurgery (*p* = 0.032, r = 0.156) (Table 2).

**Table 2:** Association of adhesion degree and operative outcomes.

Complication		No Adhesion n = 76	<50% Adhesion n = 61	50% -Buried GB n = 20	Completely Buried GB n = 0	*p*-value*	*r*
Surgery Conversion	Not Present	76 (100%)	61 (100%)	19 (95%)	0	0.032*	0.156
	Present	0	0	1 (5%)	0		
	Not Present	76 (100%)	60 (98,4%)	20 (100%)	0		
CBD Injury	Type I	0	1 (1,6%)	0	0		
	Type II	0	0	0	0	0.453	0.041
	Type III	0	0	0	0		
	Type IV	0	0	0	0		
Resurgery	Not Present	76 (100%)	61 (100%)	19 (95%)	0	0.032*	0.156
	Present	0	0	1 (5%)	0		

*significant if p < 0.05, CBD = Common Bile Duct.

## 4 Discussion

Based on our collected data, we found gallbladder adhesion is quite common (51.6% of patients who underwent laparoscopic cholecystectomy) in our health institutions. We believed most of gallbladder adhesion occurs due to delayed referral of patients. The multilevel referral system in Indonesia significantly prolongs time-to-treat and hence resulting in complications such as gallbladder adhesion. The same problem is also reported in the journal about how Indonesian national health insurance's multilevel referral system prolongs time-to-treat in patients with peritonitis [10]. Adhesion usually follows after 96 h since symptom onset, and therefore early laparoscopic is always recommended to reduce the risk of conversion or difficult laparoscopic cholecystectomy [11].

Cholecystitis with gallbladder adhesion to omentum or bowel can make laparoscopic surgery difficult. Dissection of the adhesion using blunt or sharp technique poses risk of organ perforation or organ injury. The use of cauterization during laparoscopic surgery also poses risk of immediate injury or delayed ischemic injury [12]. The adhesion of gallbladder, which most commonly involves fundus, to the omentum and other organs is considered class I difficulty which has very minimal conversion rate to open surgery. The time for surgery procedure for this difficulty class is also lowest compared to other classes difficulty that involves adhesion in Calot's triangle, adhesion of the gallbladder bed, and other intraabdominal adhesion [6].

Increasing difficulty to access the organ, ongoing inflammation and massive adhesion undoubtedly will increase the difficulty of the laparoscopic surgery and hence the time taken for the surgical procedure becomes longer [7]. One of the most alarming complications that can occur during difficult LC is biliary tract injury. In difficult LC, bile duct injury can occur especially common due to tight adhesion around the neck of the gallbladder [11]. Furthermore, errors during dissection around Calot's triangle, such as misidentification of anatomy and inability to identify injuries, are common technical factors that contribute to biliary injury. Peri-operative bleeding is also linked to an increased risk of significant bile duct injuries during LC [13,14]. Our study shows that only one subject (with <50% adhesion) had CBD injury due to short cystic duct which lies parallel and very closely to the CBD. We believed that this case of CBD is not related to the degree of adhesion. Our statistical analysis also shows that CBD injury has no significant association with degree of adhesion. We believed that this analysis can be further elaborated with bigger sample size to get a more accurate association.

In our study, one patient (in 50% - buried GB group) underwent conversion to open cholecystectomy because of massive adhesion between Hartmann pouch and Calot's triangle. During the laparoscopic surgery, it is difficult to differentiate CBD, cystic duct, and cystic arteries due to the massive adhesion. The laparoscopic surgery made no progress after 30 min and therefore conversion to open surgery is done. Our statistical analysis shows that degree of adhesion has significant correlation with the conversion to open surgery. The analysis shows that degree of GB adhesion has a significance, albeit low degree of correlation, for conversion to open surgery. This aligned with many previous studies which stated that GB adhesion is significant risk factor for conversion surgery [[15], [16], [17]].

Gallbladder adhesion also influence the decision to perform subtotal cholecystectomy instead of total cholecystectomy. Higher degree of adhesion, inflammation, and fibrosis significantly increases the risk of complication, thus resulting in difficult cholecystectomy. Subtotal cholecystectomy is considered in these difficult cases especially when the anomaly involves Calot's triangle [18,19]. Subcostal cholecystectomy has been reported as a reliable cholecystectomy procedure while preventing bile duct injury and avoiding conversion to open cholecystectomy [18]. However, in rare cases, the residue of gallbladder after cholecystectomy can still form gallbladder stones which will cause recurrence of prior symptoms (which is commonly known as post-cholecystectomy syndrome). Redo cholecystectomy can be considered for these cases, Previous studies reported the median time for redo cholecystectomy after the initial procedure varies greatly, ranging from 24 months to 60 months [18]. In the end, resurgery is required to put an end to post-cholecystectomy syndrome, and laparoscopic surgery is proven safe and reliable for complete removal of remnants of the gallbladder and gallbladder stones. In our study, the only one case of resurgery (after total laparoscopic cholecystectomy) was due to subhepatal hematoma which occurred 3 weeks after cholecystectomy. This hematoma was caused by a detached metal clip that was used during the surgery. The dislodged clip may be due to the tissue oedema around the Calot's triangle caused by the massive adhesion. We believed that this is coincidental and a rare occurrence. However, the statistical analysis shows that degree of GB adhesion has a significance, albeit low degree of correlation, for resurgery.

## 5 Conclusions

Gallbladder adhesion is considered to be commonly found in laparoscopic procedures. In our study, the degree of GB adhesion has low degree of correlation with conversion, CBD injury and resurgery. This study also showed that patients with high degree of gallbladder adhesion are still eligible for laparoscopic procedure performed by an experienced surgeon and therefore does not require immediate open surgery. A study with higher number of subjects is needed to further elaborate our findings.

## Ethical approval

This study was approved by the Institutional Review Board of the Faculty of Medicine, Public Health and Nursing, Universitas Gadjah Mada/Dr. Sardjito Hospital, Yogyakarta, Indonesia (KE/FK/0796/EC/2018).

## Sources of funding

The authors declare that this study had no funding source.

## Author contribution

Adeodatus Yuda Handaya conceived the study and approved the final draft. Aditya Rifqi Fauzi drafted the manuscript. Victor Agastya Pramudya Werdana, Ahmad Shafa Hanif, Joshua Andrew, Kevin Radinal Tjendra, and Azriel Farrel Kresna Aditya critically revised the manuscript for important intellectual content. Adeodatus Yuda Handaya, Aditya Rifqi Fauzi, Victor Agastya Pramudya Werdana, Ahmad Shafa Hanif, Joshua Andrew, Kevin Radinal Tjendra, and Azriel Farrel Kresna Aditya facilitated all project-related tasks.

## Consent

Not applicable.

## Registration of research studies

Research Repository Faculty of Medicine, Public Health and Nursing, Universitas Gadjah Mada.

Register ID: 202105099.

## Guarantor

Adeodatus Yuda Handaya.

## Provenance and peer review

Not commissioned, externally peer-reviewed.

## Declaration of competing interest

No potential conflict of interest relevant to this article was reported.
